# Evaluating risk factors affecting chronic wound healing: a comprehensive analysis using logistic regression and neural network models

**DOI:** 10.3389/frhs.2026.1889745

**Published:** 2026-08-21

**Authors:** FengMei Zhang, Jie Cui, Hui Li, Yuping Ran, Meifeng Xu

**Affiliations:** 1 Department of Oncology, Chongqing University Qianjiang Hospital, Qianjiang Central Hospital of Chongqing, Chongqing, China; 2 Department of Nursing, Chongqing University Qianjiang Hospital, Qianjiang Central Hospital of Chongqing, Chongqing, China

**Keywords:** binary logistic regression, chronic wound, multilayer perceptron neural network, risk factors, wound healing

## Abstract

**Objective:**

Chronic wounds impose immense burdens and suffering on patients, with healing governed by a complex blend of physiological, social, and psychological components. This study aimed to comprehensively evaluate risk factors affecting chronic wound healing by integrating traditional statistical methods with machine learning techniques.

**Methods:**

We retrospectively collected demographic and clinical data from 232 chronic wound patients treated at our hospital. The Mann-Whitney U test was employed for univariate comparisons of continuous variables, while categorical variables were analyzed using the chi-square or Fisher's exact test, and ordinal variables were assessed with the Cochran-Armitage trend test. Statistically significant parameters were subsequently incorporated into binary logistic regression and multilayer perceptron neural network (MLP) models.

**Results:**

Univariate analysis identified seven factors significantly associated with wound prognosis: number of concurrent wounds (*P* = 0.020), wound size (*P* = 0.043), pain score (*P* = 0.005), intervention modalities (*P* for trend = 0.005), NSAIDs use (*P* = 0.010), hemoglobin (*P* = 0.005), and albumin (*P* = 0.001). In the multivariable logistic regression model (event = good prognosis), albumin (adjusted OR=1.101, 95% CI: 1.024 -1.183, *P* = 0.010) and intervention with two modalities (OR=4.775, 95% CI: 1.215 -18.762, *P* = 0.025) or three modalities (OR=6.360, 95% CI: 1.404 -28.797, *P* = 0.016) were independently associated with good prognosis. The MLP model's AUC was recorded at 0.771 (95% CI: 0.710 -0.832), with wound size (100%) and albumin (69.3%) showing the highest normalized importance. The DeLong test showed no significant difference between the logistic regression and MLP models (*P* = 0.907).

**Conclusion:**

Albumin levels and multimodal interventions were independently associated with favorable chronic wound prognosis, while wound size and albumin emerged as the most influential predictors in the neural network model. These findings underscore the importance of nutritional status and comprehensive wound management, although causal inference is limited by the retrospective design.

## 1 Introduction

Chronic wounds, defined as persistent skin injuries that fail to heal within four weeks, represent a significant global health challenge. They are frequently associated with underlying conditions such as diabetes, pressure ulcers, venous ulcers, arterial ulcers, and trauma, profoundly affecting patients’ physical health, emotional well-being, social interactions, lifestyle, and economic stability (1). In China, the economic burden of chronic wounds is substantial. In developed regions, the average treatment cost for a chronic wound patient reaches 12,055.4 ± 9,206.3 RMB, with diabetic foot ulcers averaging 9,910.9 RMB (2). In Sichuan Province, the average hospitalization expense is 6,500.18 RMB, and diabetic patients with one or more ulcers incur a median per capita expense of 8,399.13 RMB, which far exceeds the average annual disposable income of 20,580 RMB in that region (3). This disparity places a heavy economic burden on patients in less-developed areas. The healing process for chronic wounds can be prolonged, often spanning months or even years, further exacerbating the demand for medical resources and the financial strain on patients’ families.

Epidemiological patterns of chronic wounds differ according to the degree of economic advancement in an area and the availability of medical facilities. Urban areas benefit from higher treatment rates owing to a wealth of medical facilities, whereas rural areas face challenges due to limited resources (3). Evaluating chronic wounds requires consideration of the intricate interplay among physiological, social, and psychological factors (4). Moreover, the multitude of factors influencing healing rates necessitates appropriate severity adjustments to develop effective quality indicators.

Despite extensive research, a significant gap remains in comprehensively identifying and evaluating the multifaceted risk factors affecting chronic wound healing, particularly through the integration of diverse patient characteristics and advanced analytical methodologies. Traditional statistical approaches, while valuable, may not fully capture the complex, non-linear relationships inherent in biological processes. The integration of demographic, clinical, and biochemical markers with machine learning for developing predictive models for chronic wound outcomes is underexplored. This investigation intends to address this gap with investigating a broad spectrum of factors. By employing a retrospective cohort design and integrating univariate comparisons, binary logistic regression, and multilayer perceptron (MLP) models, this research seeks to identify factors associated with chronic wound healing outcomes and evaluate the diagnostic performance of these models. The insights gained will provide valuable evidence for clinicians to devise personalized treatment strategies, thereby enhancing chronic wound healing and improving patient outcomes.

## 2 Methods

### 2.1 Study setup and involved individuals

The study, which was a retrospective cohort, was carried out at Chongqing University Qianjiang Hospital. Data were collected from 232 chronic wound patients treated between January 2023 and June 2024. The inclusion criteria specified wounds of any origin that persisted for at least four weeks. Patients with no wounds, wounds of less than four weeks’ duration, or incomplete core records that could not be supplemented by telephone follow-up were excluded. Due to the retrospective nature of the study, informed consent was not required, and the Ethics Committee of our hospital approved this study (*N*o: CQSQJZXYY-2025162).

### 2.2 Outcome definition

The primary outcome was wound-healing prognosis assessed at the end of the follow-up period. Patients were classified into good or poor prognosis groups according to the healing outcomes observed at their most recent clinical visit. A “good prognosis” was defined as complete wound healing or clinically documented improvement (≥ 50% reduction in wound area) without recurrence during the follow-up period, and a “poor prognosis” was defined as the absence of healing, < 50% area reduction, wound deterioration, or recurrence. The minimum follow-up period was 12 weeks; patients with shorter follow-up were excluded. In the logistic regression and MLP models, good wound prognosis (Outcome = 2) was modeled as the event. An OR exceeding 1 signifies an increased likelihood of a good prognosis, whereas an OR below 1 signifies an increased likelihood of a poor prognosis.

### 2.3 Data collection

Data were sourced from the medical records of both hospitalized and outpatient patients, encompassing comprehensive patient-level information: age, gender, urban/rural residence, education level, wound location, wound etiology, number of concurrent wounds, presence of wound fistulas, duration and size of wounds, pain scores, comorbid diseases, smoking and drinking status, mood disorders, intervention modalities, non-steroidal anti-inflammatory drugs (NSAID) use, hemoglobin, and serum albumin levels. To ensure authenticity and accuracy, data editing and entry were conducted by two wound-healing experts, with missing information supplemented through telephone follow-up.

Education levels were classified as no degree, primary school, junior high school, high school diploma, and college degree or above. The primary causes of chronic wounds included diabetic foot ulcer (DFU), surgical ulcer (SU), venous leg ulcer (VLU), traumatic skin ulcer (TSU), pressure ulcer (PU), and special infectious ulcer (SIU); owing to small cell counts, TSU, PU, and SIU were combined into an “Other etiologies” category for analysis. Comorbidities included diabetes, hypertension, gout, varicose veins, limb deformities, and malignant tumors. Intervention modalities were coded as none, one modality, two modalities, three modalities, and four or more modalities, encompassing routine dressing changes, surgical procedures, infection management, treatment of underlying diseases, laser therapy, and nutritional support. Emotional disorders primarily consisted of anxiety, fatigue, and loss of interest (5). Pain was evaluated using the numerical rating scale (NRS). Wound size was measured in cm^2^, the number of concurrent wounds was classified as ≤3 and >3, and the presence of fistulas was recorded. Hemoglobin and serum albumin levels were recorded at their lowest during the patient's visit for chronic wounds and are reported in g/L.

### 2.4 Statistical analysis

All statistical analyses were conducted using SPSS 29.0.1.0 (IBM, Armonk, NY). The Mann–Whitney U test was used to compare continuous variables, which were evaluated for normality and displayed as median (IQR) due to their skewed distributions. The chi-square test or Fisher's exact test was used to compare categorical variables, which are displayed as *n* (%) when expected frequencies were under 5. Ordinal variables (education level and intervention modalities) were additionally evaluated with the Cochran-Armitage trend test (linear-by-linear association). Two-sided *P* < 0.05 was considered statistically significant.

All variables with *P* < 0.05 in the univariate analysis were entered simultaneously into a multivariable binary logistic regression model to identify factors independently associated with good wound prognosis. Nominal categorical variables were entered as dummy variables with explicitly defined reference categories (intervention: none; concurrent wounds: ≤3; NSAIDs: no). Good wound prognosis (Outcome = 2) was modeled as the event. Adjusted odds ratios (ORs) with 95% confidence intervals (CIs) and *P* values are used to report results, and model calibration was checked using the Hosmer-Lemeshow goodness-of-fit test, and overall classification accuracy was reported at a cutoff of 0.5.

The variance inflation factor (VIF) was used to assess multicollinearity among predictors, with a VIF greater than 10 suggesting significant collinearity (see Supplementary Table 1). Influential observations were assessed using Cook's distance (Supplementary Table 2). Sensitivity analyses were performed by (i) excluding the 10 patients with >3 concurrent wounds (*N* = 222) and (ii) excluding the 3 patients with wound size >100 cm^2^ (*N* = 229), and the stability of OR estimates was examined (Supplementary Table 3). Because the “>3 concurrent wounds” category was rare (*n* = 10, 4.3%) and produced an unstable estimate under standard maximum likelihood estimation, Firth's bias-reduced penalized logistic regression was additionally applied to this model, and the results were compared with the standard model (Supplementary Table 4).

### 2.5 Multilayer perceptron neural network model

A multilayer perceptron (MLP) neural network was constructed using the SPSS Neural Networks module. Seven variables found to be significant in the univariate analysis served as input features. The network architecture comprised an input layer of 7 neurons, two hidden layers of 8 and 4 neurons with rectified linear unit (ReLU) activation, and a single sigmoid output neuron for binary classification. The model was trained using the Adam optimizer with binary cross-entropy loss, L2 regularization (*α* = 0.1), a learning rate of 0.001, a batch size of 32, and a maximum of 500 epochs with early stopping (patience = 20). Continuous input variables were standardized using z-score transformation. The dataset was split into a training set (*n* = 160, 69.0%) and a test set (*n* = 72, 31.0%), and 10-fold cross-validation with a fixed random seed was used to evaluate model stability. The classification threshold was set at 0.5. Variable importance was estimated by the SPSS Neural Networks module based on sensitivity analysis and reported as normalized importance (the most important variable set to 100%). The area under the receiver operating characteristic curve (AUC) was utilized to assess the model's performance. Because the SPSS MLP output does not provide a standard error or CI for the AUC, the 95% CI was estimated using the Hanley-McNeil formula. The best cutoff was identified using the Youden index, and the DeLong test was used to compare the AUCs of logistic regression and MLP models derived from the same cohort.

## 3 Results

### 3.1 Baseline characteristics

Out of the 232 patients in this study, 110 (47.4%) were categorized as having a poor prognosis, while 122 (52.6%) were categorized as having a good prognosis. The majority of patients resided in rural areas (*n* = 203, 87.5%). Educational attainment was low: the most prevalent level was primary school (*n* = 119, 51.1%), and only 12 individuals (5.2%) had completed high school or above. Comorbidities were present in 216 subjects (93.1%). Lifestyle factors included 72 smokers (31.0%) and 60 individuals (25.9%) who consumed alcohol. A total of 92 patients (39.7%) reported NSAID use, and 141 (60.8%) experienced mood disorders. The median age was 68 years (IQR 61–75) in the poor prognosis group and 65 years (IQR 58–73) in the good prognosis group, with no significant difference between groups (*P* = 0.240). Gender, residence, smoking status, drinking status, comorbidities, and mood disorders were also not significantly different between groups (Table 1).

**Table 1:** Baseline characteristics of patients stratified by wound prognosis.

Characteristic	Poor prognosis (*n* = 110)	Good prognosis (*n* = 122)	*P* value
Demographic and socioeconomic characteristics			
Gender, *n* (%)			0.718
Male	47 (42.7%)	55 (45.1%)	
Female	63 (57.3%)	67 (54.9%)	
Residence, *n* (%)			0.693
Rural	95 (86.4%)	108 (88.5%)	
Urban	15 (13.6%)	14 (11.5%)	
Education level, *n* (%)			*P* for trend = 0.082
No degree	37 (33.6%)	28 (23.0%)	
Primary school	55 (50.0%)	64 (52.5%)	
Junior high school	12 (10.9%)	24 (19.7%)	
High school diploma and above	6 (5.5%)	6 (5.0%)	
Age, years, median (IQR)	68 (61, 75)	65 (58, 73)	0.240
Wound characteristics			
Wound location, *n* (%)			0.389
Foot	46 (41.8%)	37 (30.3%)	
Lower limb	37 (33.6%)	53 (43.4%)	
Upper limb	7 (6.4%)	10 (8.2%)	
Trunk	13 (11.8%)	16 (13.1%)	
Maxillofacial	7 (6.4%)	6 (4.9%)	
Wound etiology, *n* (%)			0.138
DFU	15 (13.6%)	10 (8.2%)	
SU	72 (65.5%)	71 (58.2%)	
VLU	18 (16.4%)	31 (25.4%)	
Other etiologies (TSU + PU + SIU)	5 (4.5%)	10 (8.1%)	
Number of concurrent wounds, *n* (%)			0.020
≤ 3 wounds	109 (99.1%)	113 (92.6%)	
> 3 wounds	1 (0.9%)	9 (7.4%)	
Chronic wound fistula, *n* (%)			0.760
No	104 (94.5%)	117 (95.9%)	
Yes	6 (5.5%)	5 (4.1%)	
Wound duration, months, median (IQR)	6 (2, 24)	3 (2, 12)	0.083
Wound size, cm^2^, median (IQR)	9 (4, 20)	6 (3, 15)	0.043
Pain score, median (IQR)	3 (2, 4)	3 (2, 4)	0.005
Lifestyle and comorbidities			
Comorbid diseases, *n* (%)			0.830
No	8 (7.3%)	8 (6.6%)	
Yes	102 (92.7%)	114 (93.4%)	
Smoking status, *n* (%)			0.372
No	79 (71.8%)	81 (66.4%)	
Yes	31 (28.2%)	41 (33.6%)	
Drinking status, *n* (%)			0.982
No	82 (74.5%)	90 (73.8%)	
Yes	28 (25.5%)	32 (26.2%)	
Mood disorders, *n* (%)			0.720
No	45 (40.9%)	46 (37.7%)	
Yes	65 (59.1%)	76 (62.3%)	
Treatment characteristics			
Intervention modalities, *n* (%)			*P* for trend = 0.005
None	8 (7.3%)	4 (3.3%)	
One modality	55 (50.0%)	30 (24.6%)	
Two modalities	28 (25.5%)	58 (47.5%)	
Three modalities	11 (10.0%)	29 (23.8%)	
≥ 4 modalities	8 (7.3%)	1 (0.8%)	
NSAIDs use, *n* (%)			0.010
No	76 (69.1%)	64 (52.5%)	
Yes	34 (30.9%)	58 (47.5%)	
Laboratory indicators			
Hemoglobin, g/L, median (IQR)	122 (108, 134)	128 (118, 136)	0.005
Albumin, g/L, median (IQR)	37 (33, 40)	39 (35, 41)	0.001

DFU, diabetic foot ulcer; SU, surgical ulcer; TSU, traumatic skin ulcer; VLU, venous leg ulcer; PU, pressure ulcer; SIU, special infectious ulcer; NSAIDs, non-steroidal anti-inflammatory drugs; IQR, interquartile range.

Categorical variables are presented as *n* (%) and compared using the chi-square test or Fisher's exact test (when expected frequency < 5). Continuous variables are presented as median (IQR) and compared using the Mann–Whitney U test. The linear-by-linear association test (Cochran–Armitage trend test) was applied to assess linear trends for ordered categorical variables. Statistically significant *P* values (< 0.05) are indicated.

### 3.2 Wound characteristics

The lower limbs (*n* = 90, 38.8%) and feet (*n* = 83, 35.8%) were the primary locations for wounds, with no notable difference in distribution across groups (*P* = 0.389). The most common wound etiologies were SU (*n* = 143, 61.6%) and VLU (*n* = 49, 21.1%); etiology distribution showed no significant variation between the groups (*P* = 0.138). Ten patients (4.3%) had more than three concurrent wounds, with a higher incidence in those with a good prognosis group (9/122, 7.4%) than in the poor prognosis group (1/110, 0.9%; *P* = 0.020). Chronic wound fistula was present in 11 patients (4.7%) and did not vary among the groups (*P* = 0.760).

The good prognosis group experienced a shorter wound duration (median 3 months, IQR 2–12) compared to the poor prognosis group (median 6 months, IQR 2–24), although this difference did not reach statistical significance (*P* = 0.083). Wound size was significantly smaller in the good prognosis group (median 6 cm^2^, IQR 3–15) than in the poor prognosis group (median 9 cm^2^, IQR 4–20; *P* = 0.043). Pain scores were similar in both groups (median 3, IQR 2–4), yet the difference was significant from a statistical standpoint (*P* = 0.005), reflecting a distributional shift rather than a difference in medians (Table 1).

### 3.3 Treatment and laboratory indicators

Intervention modalities showed a significant trend across categories (*P* for trend = 0.005): patients in the good prognosis group more frequently received two modalities (58/122, 47.5%) or three modalities (29/122, 23.8%), whereas the poor prognosis group more commonly received only one modality (55/110, 50.0%) or no intervention (8/110, 7.3%). NSAID use was significantly more frequent in the good prognosis group (58/122, 47.5%) than in the poor prognosis group (34/110, 30.9%; *P* = 0.010).

Among laboratory indicators, hemoglobin levels were significantly higher in the good prognosis group (median 128 g/L, IQR 118–136) than in the poor prognosis group (median 122 g/L, IQR 108–134; *P* = 0.005). Albumin levels were also significantly higher in the good prognosis group (median 39 g/L, IQR 35–41) than in the poor prognosis group (median 37 g/L, IQR 33–40; *P* = 0.001) (Table 1).

### 3.4 Multivariable logistic regression analysis

The seven variables significant in univariate analysis (number of concurrent wounds, wound size, pain score, intervention modalities, NSAID use, hemoglobin, and albumin) were entered simultaneously into a multivariable binary logistic regression model, with good wound prognosis modeled as the event. Albumin was independently associated with good prognosis (adjusted OR=1.101 per 1 g/L increase, 95% CI: 1.024–1.183, *P* = 0.010). Intervention modalities were significantly associated with prognosis overall (*P* < 0.001): compared with no intervention, two modalities (OR=4.775, 95% CI: 1.215–18.762, *P* = 0.025) and three modalities (OR=6.360, 95% CI: 1.404–28.797, *P* = 0.016) were associated with a higher likelihood of good prognosis. The remaining variables-including hemoglobin (OR=1.007, *P* = 0.504), >3 concurrent wounds (OR=6.565, *P* = 0.101), wound size (OR=0.983, *P* = 0.105), pain score (OR=1.341, *P* = 0.101), and NSAID use (OR=1.143, *P* = 0.750)-did not reach statistical significance in the multivariable model (Table 2).

**Table 2:** Multivariable logistic regression analysis of factors associated with good wound prognosis.

Variable	Regression coefficients					Adjusted OR (95% CI)
	*β*	SE	Wald *χ*^2^	df	*P* value	
Laboratory indicators						
Hemoglobin (g/L)	0.006	0.010	0.447	1	0.504	1.007 (0.988–1.026)
Albumin (g/L)	0.096	0.037	6.722	1	**0**.**010**	1.101 (1.024–1.183)
Wound characteristics						
Number of concurrent wounds			2.696	1	0.101	—
≤ 3 wounds (Ref.)	—	—	—	—	—	*1.00 (Reference)*
> 3 wounds	1.882	1.146	2.696	1	0.101	6.565 (0.694–62.055)
Wound size (cm^2^)	−0.017	0.011	2.629	1	0.105	0.983 (0.963–1.004)
Pain score	0.293	0.179	2.694	1	0.101	1.341 (0.945–1.903)
Treatment characteristics						
Intervention modalities			22.317	4	**<0**.**001**	—
None (Ref.)	—	—	—	—	—	*1.00 (Reference)*
One modality	0.359	0.709	0.256	1	0.613	1.432 (0.357–5.753)
Two modalities	1.563	0.698	5.015	1	**0**.**025**	4.775 (1.215–18.762)
Three modalities	1.850	0.771	5.764	1	**0**.**016**	6.360 (1.404–28.796)
≥ 4 modalities	−1.214	1.300	0.871	1	0.351	0.297 (0.023–3.799)
NSAIDs use			0.102	1	0.750	—
No (Ref.)	—	—	—	—	—	*1.00 (Reference)*
Yes	0.133	0.418	0.102	1	0.750	1.143 (0.504–2.592)
Constant	−5.976	1.481	16.276	1	**<0**.**001**	

β, regression coefficient; SE, standard error; Wald χ^2^, Wald chi-square statistic; df, degrees of freedom; OR, odds ratio; CI, confidence interval; NSAIDs, non-steroidal anti-inflammatory drugs.

Model fit statistics: Hosmer–Lemeshow goodness-of-fit χ^2^ = 6.991, df = 8, *P* = 0.538 (indicating adequate fit); overall classification accuracy = 69.8%.

Variables included in the multivariable model were those with *P* < 0.05 in the univariable analysis (Table 1): hemoglobin, albumin, number of concurrent wounds, wound size, pain score, intervention modalities, and NSAIDs use. The binary logistic regression model used good wound prognosis (Outcome = 2) as the event; therefore, adjusted OR > 1 indicates a factor associated with a higher likelihood of good prognosis, whereas OR < 1 indicates a factor associated with a higher likelihood of poor prognosis.Categorical variables with more than two levels were entered as indicator (dummy) variables with the lowest category as the reference (Ref.): intervention modalities (Ref. = None); NSAIDs use (Ref. = No); number of concurrent wounds (Ref. = ≤3 wounds). Significance:.

*P* values in bold are statistically significant (*P* < 0.05).

The Hosmer-Lemeshow goodness-of-fit test indicated satisfactory model calibration (*χ*^2^ = 6.991, df = 8, *P* = 0.538), and the overall classification accuracy was 69.8% at a cutoff of 0.5. Multicollinearity diagnostics (Supplementary Table 1) showed no problematic collinearity among the predictors; the variance inflation factors (VIF) for hemoglobin and albumin were 1.540 and 1.622, respectively (all predictors VIF < 2.0, well below the conventional threshold of 10). Consequently, the loss of statistical significance for hemoglobin after multivariable adjustment (from *P* = 0.005 in univariate analysis to *P* = 0.504) cannot be attributed to collinearity with albumin. Rather, hemoglobin's independent incremental contribution was small (adjusted OR=1.007 per g/L, 95% CI: 0.988–1.026), and its univariate association appears to be largely captured by albumin—a more comprehensive marker of nutritional and inflammatory status—together with the other prognostic factors included in the model. Sensitivity analyses (Supplementary Table 3) showed that excluding patients with >3 concurrent wounds (*N* = 222) or those with wound size >100 cm^2^ (*N* = 229) changed all OR estimates by less than 10%, confirming the robustness of the main model. Because the “>3 concurrent wounds” category was rare (*n* = 10), Firth's bias-reduced penalized logistic regression was applied (Supplementary Table 4); the OR for >3 concurrent wounds decreased from 6.565 to 4.384 and the CI width narrowed by approximately 49%, while the overall conclusions remained unchanged.

### 3.5 Predictive performance of the MLP model and model comparison

**Figure 1:**
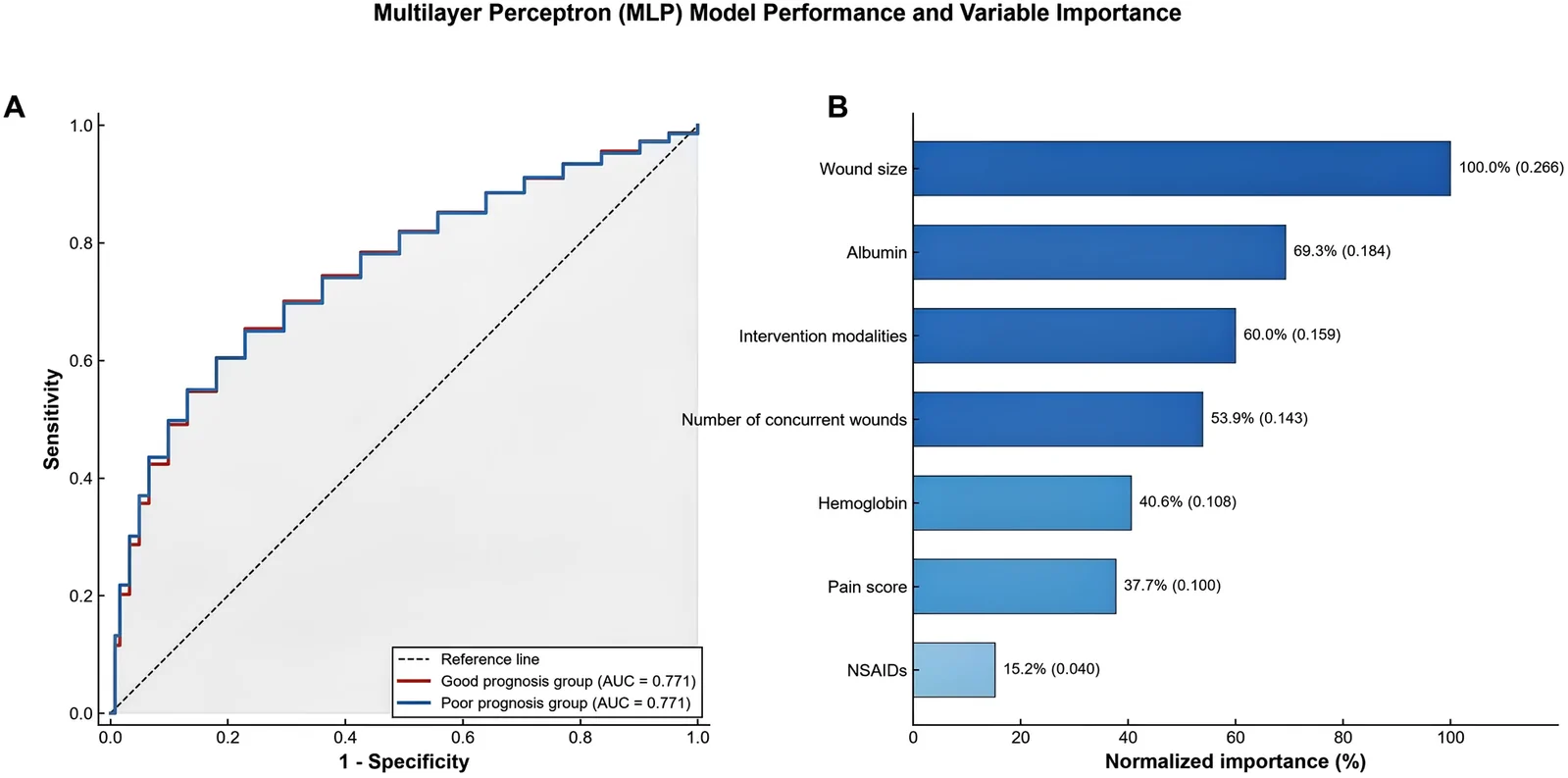
Predictive performance and variable importance of the multilayer perceptron (MLP) neural network model. **(A)** The ROC curve of the MLP model, with an AUC of 0.771 (95% CI: 0.710-0.832, estimated by the Hanley-McNeil method). At the optimal cutoff, the sensitivity was 0.604, the specificity was 0.820, and the Youden J index was 0.423. **(B)** Normalized importance of the seven input variables: wound size 100%, albumin 69.3%, intervention modalities 60.0%, number of concurrent wounds 53.9%, hemoglobin 40.6%, pain score 37.7%, and NSAID use 15.2%.

The MLP neural network incorporated the seven variables significant in univariate analysis. Model performance was assessed using a training set of 160 samples (69.0%) and a test set of 72 samples (31.0%). The training set accuracy was 73.1% and the test set accuracy was 68.1% (Table 3). The area under the ROC curve (AUC) of the MLP model was 0.771 (95% CI: 0.710–0.832, estimated by the Hanley-McNeil method). At the optimal cutoff determined by the Youden index, the MLP model achieved a sensitivity of 0.604, a specificity of 0.820, and a Youden J index of 0.423. The normalized importance rankings were as follows: wound size 100%, albumin 69.3%, intervention modalities 60.0%, number of concurrent wounds 53.9%, hemoglobin 40.6%, pain score 37.7%, and NSAID use 15.2% (Figure 1).

**Table 3:** Classification performance of the MLP neural network model in the training and test sets.

Sample	Subgroup	Accuracy
Training set (*n* = 160)	Poor prognosis	77.2%
Training set (*n* = 160)	Good prognosis	69.1%
Training set (*n* = 160)	Overall	73.1%
Test set (*n* = 72)	Poor prognosis	74.2%
Test set (*n* = 72)	Good prognosis	63.4%
Test set (*n* = 72)	Overall	68.1%

The MLP model was trained on 160 samples (69.0%) and tested on 72 samples (31.0%). Overall accuracy was 73.1% in the training set and 68.1% in the test set. The ROC AUC was 0.771 (95% CI: 0.710–0.832).

**Figure 2:**
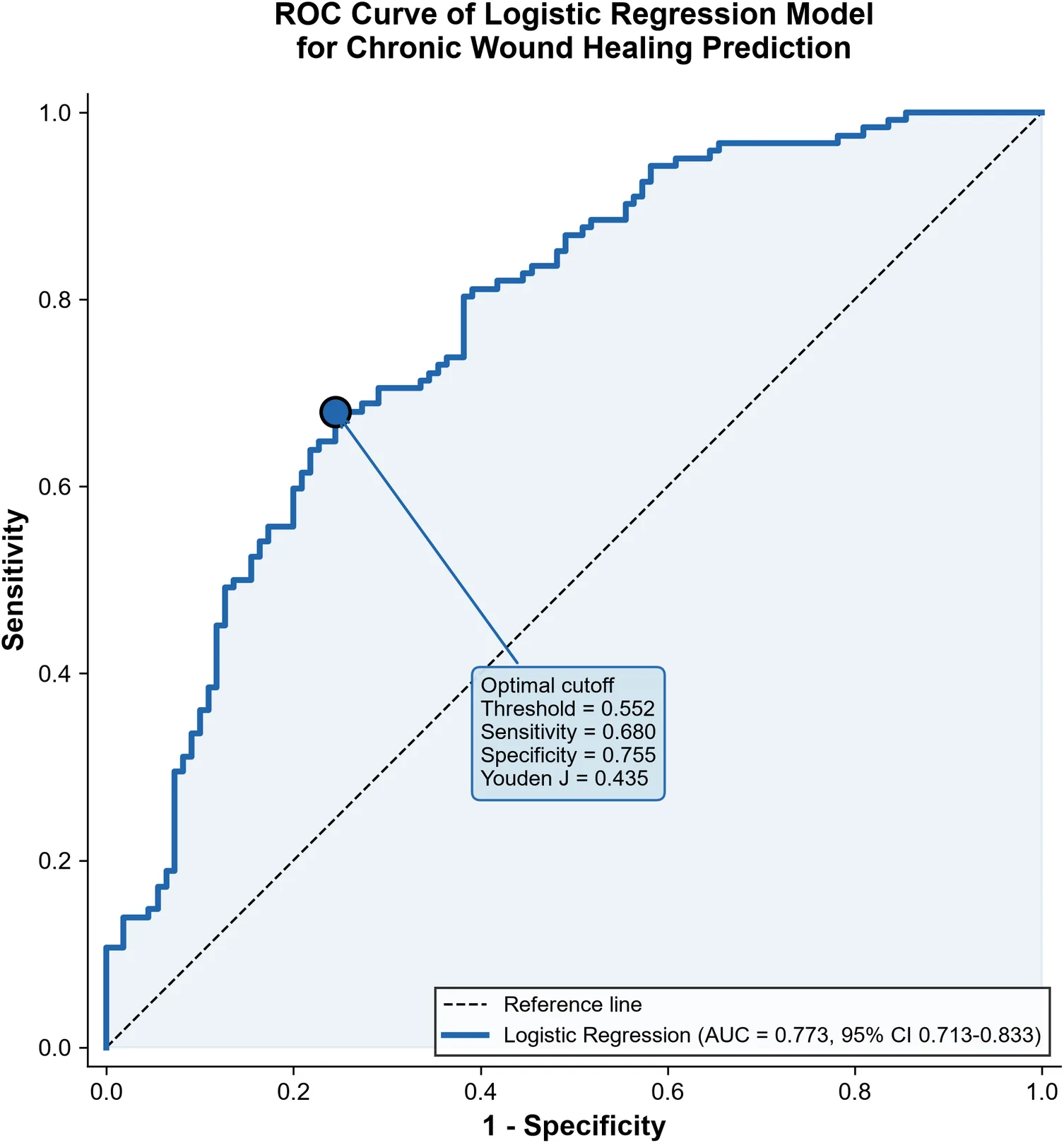
Receiver operating characteristic (ROC) curve of the binary logistic regression model for predicting chronic wound prognosis. The area under the curve (AUC) was 0.773 (95% CI: 0.713-0.833). At the optimal cutoff of 0.552 determined by the Youden index, the sensitivity was 0.680, the specificity was 0.755, and the Youden J index was 0.435. The AUC and 95% CI were obtained directly from SPSS ROC output.

**Figure 3:**
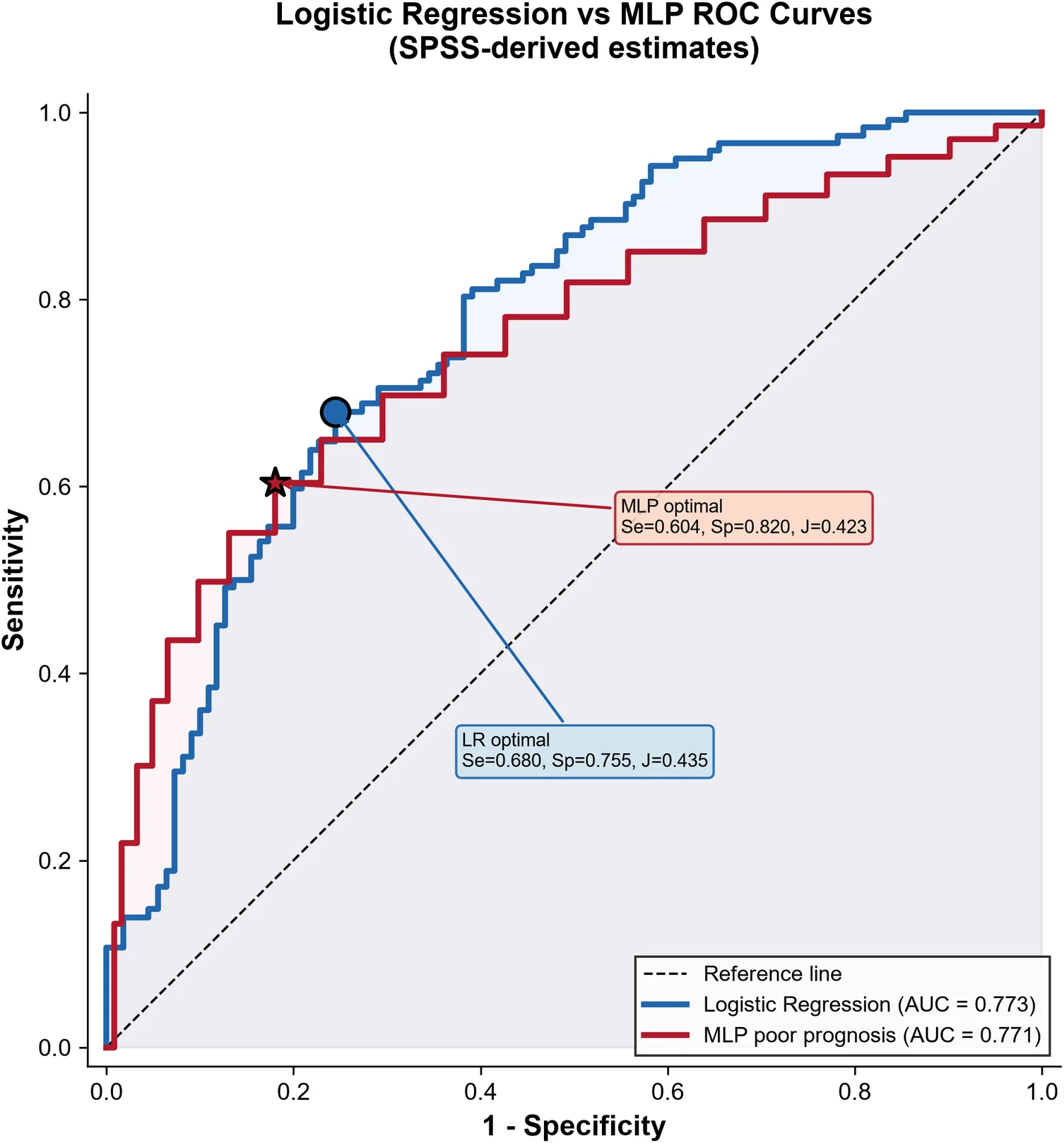
Comparison of the ROC curves of the logistic regression (AUC = 0.773) and MLP neural network (AUC = 0.771) models. The DeLong test showed no statistically significant difference between the two AUCs (*P* = 0.907), indicating comparable discriminative performance.

**Figure 4:**
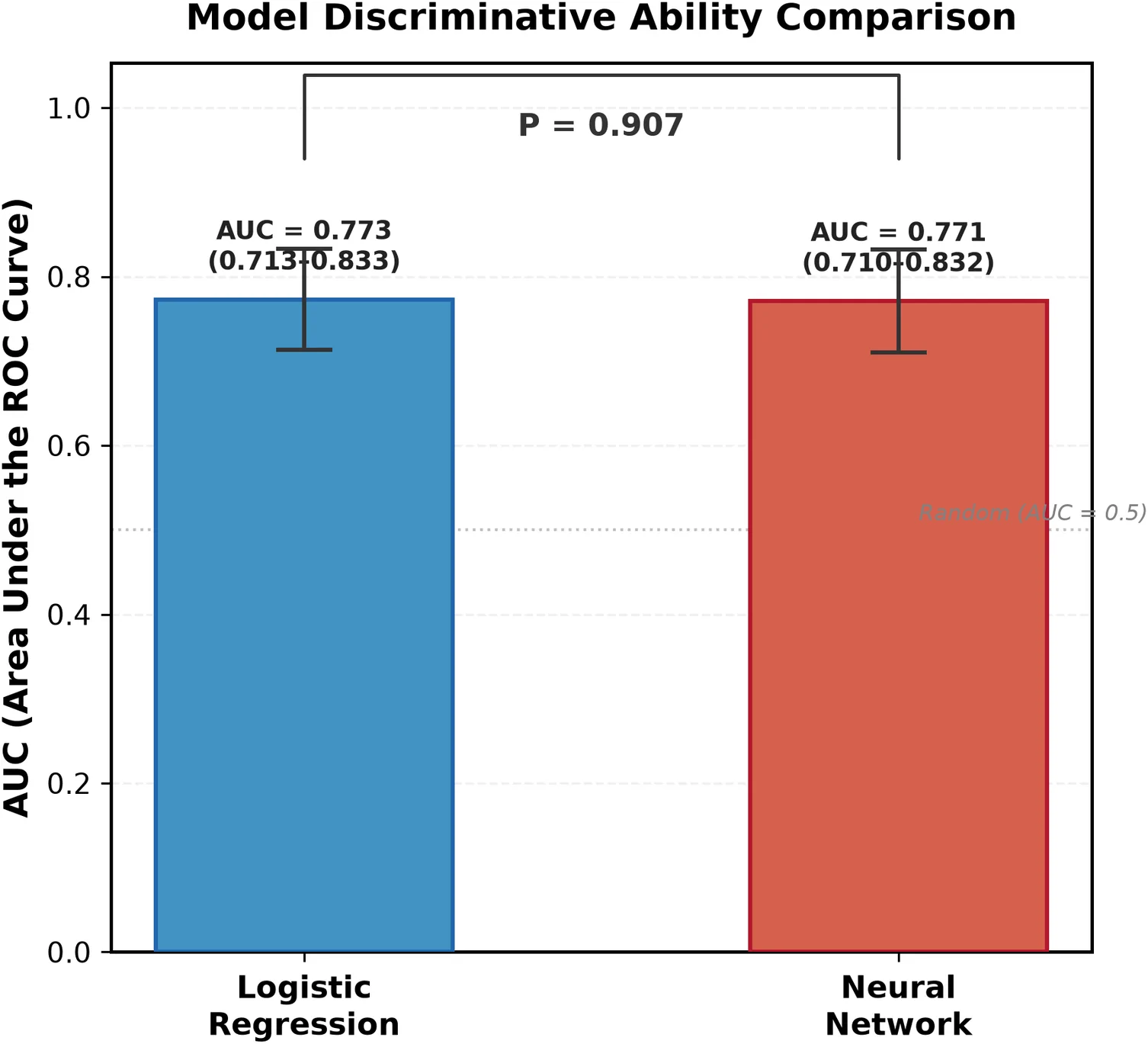
Bar chart comparing the AUCs and 95% confidence intervals of the logistic regression and MLP models. Error bars represent 95% CIs (logistic regression: 0.713-0.833; MLP: 0.710-0.832).

The logistic regression model yielded an AUC of 0.773 (95% CI: 0.713–0.833), a sensitivity of 0.680, a specificity of 0.755, and a Youden J index of 0.435 at the optimal cutoff of 0.552 (Figures 2, 3). The DeLong test showed no statistically significant difference between the AUCs of the logistic regression and MLP models (*P* = 0.907), indicating comparable discriminative performance (Figure 4). Note, however, that the logistic regression AUC was evaluated on the full cohort (*N* = 232), whereas the MLP AUC was evaluated on the held-out test set (*n* = 72); a head-to-head comparison on a common evaluation set is therefore preferable, and the non-significant DeLong result should be interpreted with this caveat in mind. Although their AUCs were similar, the two models offered complementary strengths: the logistic regression provided interpretable effect estimates (adjusted ORs), whereas the MLP quantified non-linear variable importance.

## 4 Discussion

Chronic wounds impose significant physical and mental distress on patients while also creating a substantial financial burden for both individuals and the healthcare system. These wounds often exist in a state of ongoing inflammation, with healing influenced by factors such as blood glucose levels, cellular aging, and microcirculatory disorders (6, 7). This retrospective study of 232 patients integrated univariate comparisons, multivariable logistic regression, and an MLP neural network to identify factors associated with chronic wound prognosis and to analyze the effectiveness of these two modeling approaches in distinguishing results. The principal findings were that albumin and multimodal interventions (two or three modalities) were independently associated with good prognosis, that wound size and albumin were the most influential predictors in the MLP model, and that the logistic regression and MLP models achieved comparable AUCs. Because this is a retrospective, single-center, observational study, all associations ought to be considered prognostic patterns instead of causal effects.

### 4.1 Nutritional Status: albumin and hemoglobin

Albumin was independently associated with good wound prognosis in the multivariable model (adjusted OR=1.101 per 1 g/L increase, 95% CI: 1.024–1.183, *P* = 0.010), and it was the second most important predictor in the MLP model (normalized importance 69.3%). This result supports the vital importance of nutritional status, particularly protein adequacy, in wound healing, and aligns with European guidelines for managing pressure ulcers, which emphasize nutrition and identify malnutrition as a detrimental factor, especially in elderly patients with chronic wounds (8, 9). However, serum albumin is recognized as a limited nutritional marker for predicting wound healing, as its level reflects not only nutritional status but also systemic inflammation and hepatic synthesis capacity (10). Hemoglobin was significant in univariate analysis (*P* = 0.005) but lost significance after multivariable adjustment (*P* = 0.504). Multicollinearity diagnostics (Supplementary Table 1) showed no concerning collinearity between hemoglobin and albumin (VIF = 1.540 and 1.622, respectively), as both are haematological/nutritional indicators that partly reflect overlapping nutritional and inflammatory status. Because the VIF values were far below the threshold for problematic collinearity, the attenuation of hemoglobin's effect after adjustment is better explained by its modest independent contribution relative to albumin—the stronger, more comprehensive nutritional marker—rather than by collinearity *per se*. Accordingly, albumin should be interpreted as a marker of overall nutritional and inflammatory status rather than as an isolated causal determinant, and its independent contribution should be viewed in the context of these shared nutritional pathways. Accordingly, albumin should be interpreted as a marker of overall nutritional and inflammatory status rather than as an isolated causal determinant (10), and its independent contribution should be viewed in the context of this collinearity.

### 4.2 Intervention modalities: association, not causation

Receiving two or three intervention modalities was linked to an increased probability of good prognosis compared with no intervention (OR=4.775 and 6.360, respectively). However, because this is a retrospective observational study, this association does not imply that multimodal interventions cause better healing. There is a substantial risk of confounding by indication (11): patients with more severe or complex wounds may be more likely to receive multiple interventions, while those with better access to care may receive more comprehensive treatment. The observed association may therefore reflect a combination of baseline severity, access to care, and treatment intensity rather than a direct treatment effect. We explicitly avoid claiming that multimodal interventions improve prognosis, and any clinical implication should be framed as a prognostic pattern requiring confirmation in prospective studies that adjust for baseline severity, etiology, infection status, vascular status, diabetes, adherence, and follow-up time. Finally, the apparent benefit of multimodal intervention should be interpreted primarily for the two- and three-modality groups: the ≥4-modality group was small (*n* = 9) and, contrary to the overall trend, showed a lower good-prognosis rate (1/9, 11.1%) than even the no-intervention group, which likely reflects confounding by indication or treatment-limiting disease severity in this subset rather than a true dose–response plateau.

### 4.3 Clinically paradoxical findings: pain and NSAIDs

Two findings warrant cautious interpretation. First, NSAID use was more frequent in the good prognosis group (58/122, 47.5%) than in the poor prognosis group (34/110, 30.9%), and it lost significance in the multivariable model (*P* = 0.750). Second, although pain scores were statistically different between groups (*P* = 0.005), the medians were identical (3 in both groups), and higher pain scores showed a trend toward association with good prognosis in the multivariable model (OR=1.341, *P* = 0.101). These patterns contradict a simple interpretation of pain or NSAID use as markers of worse outcome and likely reflect indication bias and confounding: patients with inflammatory symptoms may be more likely to receive NSAIDs and to have more active wound care, pain perception may differ by wound etiology (e.g., diabetic neuropathy, which causes loss of protective sensation and altered pain perception in the lower extremities) (12), and access to pain management may vary. We therefore avoid mechanistic conclusions regarding NSAIDs and pain. NSAIDs inhibit cyclooxygenase and may disrupt healing in acute wounds, whereas chronic wounds are characterized by persistent inflammation, and the net effect of NSAIDs in this setting is complex (13, 14).

### 4.4 Wound size and concurrent wounds

The group with a poor prognosis had significantly larger wound sizes in univariate analysis (median 9 vs. 6 cm^2^, *P* = 0.043) and was the most influential predictor in the MLP model (normalized importance 100%), consistent with the biological expectation that larger wounds require more extensive tissue regeneration and are more susceptible to complications, a relationship well documented in venous leg ulcer prognostic models where initial wound area is a strong predictor of healing time (15). Wound size has been repeatedly confirmed as one of the most robust prognostic markers across diverse wound etiologies (16, 17).

In the multivariable logistic regression, however, wound size did not retain independent significance (OR=0.983, *P* = 0.105), likely owing to shared variance with intervention modalities and nutritional indicators. This divergence between the logistic regression and the MLP is methodologically informative: the linear model estimates the independent effect of a single factor after adjusting for others, whereas the MLP can capture non-linear interactions between wound size and other prognostic factors, which may explain its dominant feature importance. The relationship between wound size and healing probability is unlikely to be strictly linear; rather, threshold effects may exist where wounds exceeding a critical area experience disproportionately worse outcomes due to overwhelming the regenerative capacity of the surrounding tissue. The MLP, with its hidden-layer activation functions, is well suited to model such sigmoidal or step-function relationships, whereas logistic regression assumes a log-linear relationship that may underestimate the prognostic value of wound size at the extremes of the distribution. This observation aligns with growing evidence that machine learning approaches can capture complex feature interactions in wound prognosis that traditional regression models may overlook (18, 19).

### 4.5 Model comparison and implications

The logistic regression (AUC = 0.773, 95% CI: 0.713–0.833) and MLP (AUC = 0.771, 95% CI: 0.710–0.832) models achieved comparable discriminative performance, with no significant difference by the DeLong test (*P* = 0.907). The two approaches offered complementary strengths: logistic regression provided interpretable adjusted ORs that quantify the direction and magnitude of association for each factor, whereas the MLP captured non-linear relationships and ranked variables by predictive importance. Multilayer perceptron neural networks have been increasingly applied to predict diabetic foot ulcer outcomes, including mortality and amputation risk (20, 21), supporting the feasibility of this approach in wound prognosis research. For comparison, Cho et al. reported AUCs of 0.712 and 0.717 for logistic and classification tree models, respectively, and found that wound-specific characteristics (location, area, depth, etiology) were more effective predictors than patient demographics and comorbidities (19). Our findings similarly support the importance of wound size as a predictive factor. It should be emphasized that the variable importance from the MLP reflects predictive contribution within this dataset and should not be interpreted as evidence of a causal role unless externally validated.

### 4.6 Findings not confirmed and potential explanations

This study found no statistically significant difference in prognosis across wound etiologies. Diabetic patients often receive more standardized management within specific healthcare settings, as exemplified by the International Working Group on the Diabetic Foot (IWGDF) evidence-based guidelines that standardize prevention and treatment protocols (22), which may offset the inherent challenges of diabetic wounds. In contrast, patients with other chronic wound etiologies may face barriers to accessing timely and standardized care, including economic constraints and transportation issues, resulting in fewer opportunities for essential interventions such as debridement, pressure relief, appropriate dressings, and vascular assessment. Thus, the accessibility of medical resources may be closely linked to healing outcomes. Although mood disorders (anxiety, fatigue, diminished interest) have been reported in patients with chronic wounds and may contribute to delayed healing and reduced quality of life (5), this study did not confirm such an association, possibly because the retrospective design did not adequately capture the severity of mood disorders.

### 4.7 Limitations

Several limitations exist in this study. First, the single-center, retrospective design restricts diversity and representativeness and precludes causal inference; these findings should be considered as exploratory and intended to generate hypotheses. Second, the sample size, while acceptable for an exploratory observational analysis, is modest for machine-learning purposes, and a single train/test split may yield unstable estimates; the MLP results require external validation. Third, the lack of long-term follow-up data hampers assessment of treatment efficacy and recurrence over time. Fourth, the moderate correlation between hemoglobin and albumin complicates the attribution of their independent effects: hemoglobin's univariate association was not retained after adjustment for albumin and other prognostic factors, although multicollinearity diagnostics indicated no problematic collinearity (VIF < 1.7). Fifth, residual confounding by indication (14), baseline severity, and access to care cannot be excluded in the interpretation of intervention-related associations. Sixth, the DeLong comparison of the two models’ AUCs was based on the logistic regression evaluated on the full cohort (*N* = 232) and the MLP evaluated on a held-out test set (*n* = 72); because the two AUCs were not estimated on an identical sample, the non-significant DeLong result should be interpreted cautiously. Future research should use larger, multicenter, prospective cohorts with extended follow-up, incorporate time-to-event methods (e.g., Cox regression) when follow-up duration varies, and perform external validation of the predictive models.

## 5 Conclusion

In this exploratory retrospective study, serum albumin levels and multimodal interventions (two or three modalities) were independently associated with favorable chronic wound prognosis, whereas wound size and albumin emerged as the most influential predictors in the MLP neural network model. The logistic regression and MLP models achieved comparable discriminative performance (AUC ≈ 0.77). These discoveries stress the crucial nature of nutritional status and comprehensive wound management in chronic wound care. However, owing to the single-center, retrospective design and the possibility of confounding due to indication, we consider the observed associations as prognostic patterns instead of causal effects. Future prospective, multicenter studies with long-term tracking and external validation are required to substantiate these findings and to elucidate the mechanisms underlying chronic wound healing.

## Data Availability

The datasets presented in this study can be found in online repositories. The names of the repository/repositories and accession number(s) can be found in the article/Supplementary Material.

## Supplementary material

Supplementary Tables 1–4 Are provided as separate files: S1 (multicollinearity diagnosis with VIF), S2 (influential observations by Cook's distance), S3 (sensitivity analyses), and S4 (Firth's bias-reduced penalized logistic regression).

Supplementary Figure (Neural Network Architecture) Architecture of the multilayer perceptron neural network: an input layer of 7 neurons (the seven variables significant in univariate analysis), two hidden layers of 8 and 4 neurons with ReLU activation, and a single sigmoid output neuron. The model was trained with the Adam optimizer, binary cross-entropy loss, and L2 regularization (α = 0.1) using 10-fold cross-validation.
