# Supplementary material for: Evaluating risk factors affecting chronic wound healing: a comprehensive analysis using logistic regression and neural network models
Source: Front Health Serv. 2026 Aug 21;6:1889745. doi: 10.3389/frhs.2026.1889745 (PMC13541927; doi:10.3389/frhs.2026.1889745)
Supplement: Supplementary file 1 [file Table1.docx]

**Supplementary Table S1. Multicollinearity Diagnosis for the Multivariable Logistic Regression Model**

| **Predictor Variable** | **VIF** | **Tolerance** | **Collinearity Status** |
| --- | --- | --- | --- |
| Hemoglobin (g/L) | 1.540 | 0.649 | Acceptable |
| Albumin (g/L) | 1.622 | 0.617 | Acceptable |
| Wound size (cm²) | 1.543 | 0.648 | Acceptable |
| Pain score | 1.951 | 0.513 | Acceptable |
| NSAIDs use (Yes vs No) | 1.901 | 0.526 | Acceptable |
| >3 concurrent wounds (vs ≤3) | 1.026 | 0.975 | Acceptable |
| Intervention: One modality (vs None) | 1.077 | 0.929 | Acceptable |

**Abbreviations:** VIF, variance inflation factor; NSAIDs, non-steroidal anti-inflammatory drugs.

**Note:** VIF = variance inflation factor. The multivariable binary logistic regression model included all variables with P < 0.05 in univariate analysis (Table 1): Hemoglobin, Albumin, Wound size, Pain score, Number of concurrent wounds (>3 vs ≤3), Intervention modalities (dummy coded, reference = no intervention), and NSAIDs use (Yes vs No). Good wound prognosis (Outcome = 2) was modeled as the event.

VIF interpretation: VIF < 5 = acceptable (no significant collinearity); VIF 5–10 = moderate collinearity; VIF > 10 = severe collinearity requiring attention. Tolerance = 1/VIF; values < 0.1 indicate severe collinearity.
