# Supplementary material for: Evaluating risk factors affecting chronic wound healing: a comprehensive analysis using logistic regression and neural network models
Source: Front Health Serv. 2026 Aug 21;6:1889745. doi: 10.3389/frhs.2026.1889745 (PMC13541927; doi:10.3389/frhs.2026.1889745)
Supplement: Supplementary file 2 [file Table2.docx]

**Supplementary Table S2. Influential Observations and Outliers Diagnosis for the Multivariable Logistic Regression Model**

**Part A. Summary of Influence Diagnostics**

| **Diagnostic Statistic** | **Value** |
| --- | --- |
| Total observations (N) | 232 |
| Model parameters (p) | 11 |
| Cook's distance threshold (4/N) | 0.0172 |
| Leverage threshold (2p/N) | 0.0948 |
| Standardized residual threshold | \|r\| > 2.5 |
|  |  |
| Cook's distance, maximum | 0.1377 |
| Cook's distance, mean | 0.0051 |
| Leverage, maximum | 0.3342 |
| Leverage, mean | 0.0474 |
|  |  |
| Observations exceeding Cook's distance threshold, n (%) | 12 (5.2%) |
| Observations exceeding leverage threshold, n (%) | 22 (9.5%) |
| Observations with \|Pearson residual\| > 2.5, n (%) | 1 (0.4%) |

**Part B. Top 15 Most Influential Observations (Ranked by Cook's Distance)**

| **Case No.** | **Cook's Distance** | **Leverage** | **Pearson Residual** | **Deviance Residual** | **Outcome** | **Intervention** | **>3 Wounds** | **Wound Size (cm²)** | **Albumin (g/L)** | **Hemoglobin (g/L)** |
| --- | --- | --- | --- | --- | --- | --- | --- | --- | --- | --- |
| **12** | **0.1377** | **0.3048** | -1.5498 | -1.5648 | Poor | One | Yes | 48.0 | 39.6 | 163.0 |
| **227** | **0.1058** | **0.3342** | 1.2425 | 1.3665 | Good | ≥4 | No | 1.5 | 41.9 | 135.0 |
| **221** | **0.0328** | **0.2377** | 0.9390 | 1.1244 | Good | Three | No | 100.0 | 38.2 | 155.0 |
| **175** | **0.0277** | **0.1274** | -1.3504 | -1.4409 | Poor | None | No | 1.5 | 39.9 | 137.0 |
| **137** | **0.0271** | **0.0959** | 1.5926 | 1.5894 | Good | None | No | 6.0 | 38.9 | 129.0 |
| **214** | **0.0265** | **0.3222** | 0.6452 | 0.8344 | Good | One | Yes | 56.0 | 35.8 | 84.0 |
| **135** | **0.0247** | **0.1197** | 1.3258 | 1.4244 | Good | None | No | 18.0 | 40.9 | 122.0 |
| **25** | **0.0207** | **0.1114** | 1.2690 | 1.3853 | Good | None | No | 4.0 | 41.1 | 116.0 |
| **43** | **0.0194** | 0.0774 | -1.5301 | -1.5533 | Poor | Two | No | 3.0 | 41.7 | 138.0 |
| **154** | **0.0193** | 0.0400 | -2.2130 | -1.8839 | Poor | Three | No | 9.0 | 41.2 | 161.0 |
| **203** | **0.0192** | 0.0841 | 1.4527 | 1.5065 | Good | Two | No | 20.0 | 22.1 | 111.0 |
| **45** | **0.0186** | **0.1009** | 1.2818 | 1.3942 | Good | Two | No | 3.0 | 19.8 | 118.0 |
| 226 | 0.0168 | 0.0346 | -2.2293 | -1.8903 | Poor | Three | No | 22.8 | 39.5 | 159.0 |
| 228 | 0.0168 | 0.0388 | -2.0959 | -1.8358 | Poor | Three | No | 20.0 | 37.7 | 114.0 |
| **139** | 0.0165 | 0.0260 | **-2.5712** | -2.0147 | Poor | Two | No | 4.0 | 44.9 | 163.0 |

**Abbreviations:** N, total sample size; p, number of model parameters; NSAIDs, non-steroidal anti-inflammatory drugs.

**Note:** Influence diagnostics were computed from the multivariable binary logistic regression model (Supplementary Table S1). Good wound prognosis (Outcome = 2) was modeled as the event. Part A presents summary statistics; Part B lists the 15 observations with the largest Cook's distances.

Thresholds: Cook's distance > 4/N (0.0172); Leverage > 2p/N (0.0948); |Pearson residual| > 2.5. Values exceeding thresholds are shown in bold: Cook's distance in red, leverage in orange, Pearson residual in red.

A total of 12 observations (5.2%) exceeded the Cook's distance threshold, 22 (9.5%) exceeded the leverage threshold, and 1 (0.4%) had large standardized residuals. No single observation exerted disproportionate influence on the model (maximum Cook's distance = 0.138, well below the conventional concern threshold of 1.0).

Sensitivity analysis excluding the 3 most influential observations resulted in model non-convergence due to complete separation (see Supplementary Table S3), indicating that these observations contain critical information for model stability rather than being aberrant outliers.
