# Supplementary material for: Evaluating risk factors affecting chronic wound healing: a comprehensive analysis using logistic regression and neural network models
Source: Front Health Serv. 2026 Aug 21;6:1889745. doi: 10.3389/frhs.2026.1889745 (PMC13541927; doi:10.3389/frhs.2026.1889745)
Supplement: Supplementary file 3 [file Table3.docx]

**Supplementary Table S3. Sensitivity Analyses for the Multivariable Logistic Regression Model**

| **Predictor Variable** | **Main Model OR (N=232)** | **Sensitivity Analysis 1 Exclude >3 wounds (N=222)** | **ΔOR (%)** | **OR Change** | **Sensitivity Analysis 3 Exclude Wound size >100 cm² (N=229)** | **ΔOR (%)** | **OR Change** |
| --- | --- | --- | --- | --- | --- | --- | --- |
| Hemoglobin (g/L) | 1.007 | 1.010 | +0.4% | Stable | 1.006 | -0.0% | Stable |
| Albumin (g/L) | 1.101 | 1.091 | -0.8% | Stable | 1.101 | +0.0% | Stable |
| Wound size (cm²) | 0.983 | 0.985 | +0.2% | Stable | 0.984 | +0.1% | Stable |
| Pain score | 1.341 | 1.309 | -2.4% | Stable | 1.345 | +0.3% | Stable |
| NSAIDs use (Yes vs No) | 1.143 | 1.111 | -2.8% | Stable | 1.138 | -0.4% | Stable |
| >3 wounds (vs ≤3) | 6.565 | *—* | — | Excluded | 6.380 | -2.8% | Stable |
| Intervention: One modality | 1.432 | 1.390 | -3.0% | Stable | 1.434 | +0.1% | Stable |
| Intervention: Two modalities | 4.775 | 4.596 | -3.7% | Stable | 4.753 | -0.5% | Stable |
| Intervention: Three modalities | 6.360 | 5.799 | -8.8% | Stable | 6.292 | -1.1% | Stable |
| Intervention: ≥4 modalities | 0.297 | 0.303 | +2.0% | Stable | 0.296 | -0.2% | Stable |

**Abbreviations:** OR, odds ratio; ΔOR, change in odds ratio; N, sample size; NSAIDs, non-steroidal anti-inflammatory drugs.

**Note:** Sensitivity analyses were performed to evaluate the robustness of the multivariable logistic regression model. Good wound prognosis (Outcome = 2) was modeled as the event. OR = odds ratio; ΔOR (%) = percentage change in OR relative to the main model.

Main model: N = 232, including all 7 predictors with P < 0.05 in univariate analysis (Table 1). All predictors were entered simultaneously. Intervention modalities were dummy coded with "no intervention" as the reference.

Sensitivity Analysis 1: Excluded 10 observations with >3 concurrent wounds (retaining only ≤3 wounds cases, N = 222). The ">3 wounds" predictor was removed from this model. All remaining ORs changed by <10%, confirming that the small subgroup of >3 wounds did not disproportionately influence the main model estimates.

Sensitivity Analysis 2 (not shown): Excluded the 3 most influential observations by Cook's distance. This analysis resulted in model non-convergence due to complete separation (the "≥4 modalities" category became perfectly separated). This indicates that these 3 observations contain critical information for model stability rather than being aberrant outliers, and their exclusion is not statistically appropriate.

Sensitivity Analysis 3: Excluded 3 observations with wound size >100 cm² (N = 229). All ORs changed by <3%, demonstrating that extreme wound size values did not influence the model conclusions.

OR change interpretation: "Stable" = |ΔOR| < 10% (green); "Notable" = |ΔOR| ≥ 10% (red). Overall, sensitivity analyses confirmed the robustness of the main model conclusions. The independent protective effects of Albumin (OR ≈ 1.10, P < 0.05) and Intervention modalities (two and three modalities vs none, OR ≈ 4.6–6.4, P < 0.05) remained consistent across all sensitivity analyses.
