# Supplementary material for: Evaluating risk factors affecting chronic wound healing: a comprehensive analysis using logistic regression and neural network models
Source: Front Health Serv. 2026 Aug 21;6:1889745. doi: 10.3389/frhs.2026.1889745 (PMC13541927; doi:10.3389/frhs.2026.1889745)
Supplement: Supplementary file 4 [file Table4.docx]

**Supplementary Table S4.** Comparison of Standard Maximum Likelihood Logistic Regression and Firth’s Bias-Reduced Penalized Logistic Regression for Identifying Factors Associated with Good Wound Prognosis

| **Variable** | **Standard MLR** | | | | **Firth Penalized Regression** | | | | **Comparison** |
| --- | --- | --- | --- | --- | --- | --- | --- | --- | --- |
|  | **β** | **SE** | **Adjusted OR (95% CI)** | **P** | **β** | **SE** | **Adjusted OR (95% CI)** | **P** | **OR Change (%)** |
| ***Laboratory indicators*** |  |  |  |  |  |  |  |  |  |
| Hemoglobin (g/L) | 0.0065 | 0.0097 | 1.007 (0.988–1.026) | 0.504 | 0.0059 | 0.0095 | 1.006 (0.987–1.025) | 0.534 | **-0.1%** |
| **Albumin (g/L)** | 0.0958 | 0.0369 | **1.101 (1.024–1.183)** | **0.010** | 0.0900 | 0.0362 | **1.094 (1.019–1.175)** | **0.013** | **-0.6%** |
| ***Wound characteristics*** |  |  |  |  |  |  |  |  |  |
| Wound size (cm²) | -0.0172 | 0.0106 | 0.983 (0.963–1.004) | 0.105 | -0.0148 | 0.0102 | 0.985 (0.966–1.005) | 0.144 | **+0.2%** |
| Pain score | 0.2933 | 0.1787 | 1.341 (0.945–1.903) | 0.101 | 0.2772 | 0.1757 | 1.319 (0.935–1.862) | 0.115 | **-1.6%** |
| >3 concurrent wounds vs ≤3 (Ref.) | 1.8817 | 1.1461 | 6.565 (0.694–62.058) | 0.101 | 1.4780 | 1.0090 | 4.384 (0.607–31.678) | 0.143 | **-33.2% ←** |
| ***Treatment characteristics*** |  |  |  |  |  |  |  |  |  |
| NSAIDs use: Yes vs No (Ref.) | 0.1333 | 0.4179 | 1.143 (0.504–2.592) | 0.750 | 0.1232 | 0.4128 | 1.131 (0.504–2.540) | 0.765 | **-1.0%** |
| Intervention: One vs None (Ref.) | 0.3593 | 0.7094 | 1.432 (0.357–5.754) | 0.613 | 0.2922 | 0.6995 | 1.339 (0.340–5.276) | 0.676 | **-6.5%** |
| **Intervention: Two vs None (Ref.)** | 1.5635 | 0.6982 | **4.775 (1.215–18.762)** | **0.025** | 1.4514 | 0.6878 | **4.269 (1.109–16.438)** | **0.035** | **-10.6%** |
| **Intervention: Three vs None (Ref.)** | 1.8500 | 0.7706 | **6.360 (1.404–28.797)** | **0.016** | 1.7040 | 0.7568 | **5.496 (1.247–24.225)** | **0.024** | **-13.6%** |
| Intervention: ≥4 vs None (Ref.) | -1.2137 | 1.3002 | 0.297 (0.023–3.799) | 0.351 | -0.9228 | 1.1885 | 0.397 (0.039–4.083) | 0.438 | **+33.8%** |
| **Constant** | -5.9762 | 1.4813 | **0.003 (0.000–0.046)** | **<0.001** | -5.5794 | 1.4509 | **0.004 (0.000–0.065)** | **<0.001** | **+48.7%** |

**Abbreviations:** *MLR, maximum likelihood regression; OR, odds ratio; CI, confidence interval; SE, standard error; β, regression coefficient; NSAIDs, non-steroidal anti-inflammatory drugs; Ref., reference category.*

**Note:** All variables with P < 0.05 in univariate analysis (Table 1) were entered into the multivariate binary logistic regression model. Good wound prognosis (Outcome = 2) was modeled as the event; OR > 1 indicates a higher likelihood of good prognosis, whereas OR < 1 indicates a higher likelihood of poor prognosis. Standard MLR refers to standard maximum likelihood estimation. Firth penalized regression applies Firth’s bias-reduction correction (Firth, 1993; Heinze & Schemper, 2002), which modifies the score function with a Jeffreys prior penalty. This method is specifically designed for rare-event data and separation problems, producing finite, more conservative estimates with narrower confidence intervals for sparse categories. The >3 concurrent wounds category (highlighted in red, marked with ←) contained only 10 cases (4.3% of the sample: 9 good prognosis, 1 poor prognosis). Under standard MLR, the OR was 6.565 (95% CI 0.694–62.058, P = 0.101) with a very wide CI (width = 61.36). Firth’s regression reduced the OR to 4.384 (95% CI 0.607–31.678, P = 0.143) and narrowed the CI by 49.4% (width = 31.07), confirming that the estimate was unstable under standard MLR but became more reliable under penalized estimation. Both methods consistently identified Albumin and Intervention with two or three modalities as significant independent factors for good prognosis (P < 0.05), and >3 concurrent wounds remained non-significant in both approaches. Statistically significant variables (P < 0.05) are presented in bold.

Model fit — Standard MLR: log-likelihood = -130.256, AIC = 282.513, BIC = 320.427; Firth: penalized log-likelihood = -110.475, AIC = 282.908, BIC = 320.822. Both models converged successfully.
